# Complete mitochondrial genome of the whitefly *Aleyrodes shizuokensis* Kuwana (Hemiptera: Aleyrodidae), new record from Chinese mainland

**DOI:** 10.1080/23802359.2020.1869617

**Published:** 2021-02-08

**Authors:** Teng Lei, Yu-Wei Zhong, Yin-Quan Liu

**Affiliations:** Ministry of Agriculture Key Lab of Molecular Biology of Crop Pathogens and Insects, Institute of Insect Sciences, Zhejiang University, Hangzhou, China

**Keywords:** Chinese mainland, mitochondrial genome, phylogeny, whitefly

## Abstract

The complete mitochondrial genome was determined for the whitefly *Aleyrodes shizuokensis* (Hemiptera: Aleyrodidae), the first record from Chinese mainland. The mitochondrial genome is 16,687 bp in length and contains 13 protein-coding genes (PCGs), 22 transfer RNAs, and two ribosomal RNAs. The overall base composition is 33.8% A, 47.0% T, 12.2% G, and 7.0% C. All PCGs start with ATN codon. *COX1* ends with a T, and the other 12 PCGs use TAA or TAG as the stop codon. Gene arrangement of the 13 PCGs is identical to that of the giant whitefly *Aleurodicus dugesii* and greenhouse whitefly *Trialeurodes vaporariorum*. The resultant Bayesian inference and maximum-likelihood trees based on the sequence data of 13 PCGs support its close relationship with sugarcane whitefly *Neomaskellia andropogonis*.

The whitefly *Aleyrodes shizuokensis* Kuwana 1911 (Hemiptera: Aleyrodidae) was recorded from Japan, Hawaii, India and Taiwan (Takahashi 1935; Paulson and Kumashiro 1985; David and Jesudasan 1989). In this work, *A. shizuokensis* was first found in Chinese mainland. In insect taxonomy, morphological characteristics and DNA barcodes are used for species identification and phylogenetic analysis. Chen et al. (2007) described the puparium and adults of *A. shizuokensis*, providing sufficient morphological characteristics of this species. Whereas, only two DNA barcodes of *A. shizuokensis* are deposited in GenBank nucleotide sequence database, i.e. 16S ribosomal RNA (GQ867759) and cytochrome c oxidase subunit I (GQ867730). Mitochondrial genomes have been broadly used in phylogenetic analysis (Ma et al. 2012) and are powerful means for inferring ancient evolutionary relationships (Boore 1999). So far, the *A. shizuokensis* mitochondrial genome and DNA barcode-based phylogenetic analysis have not been reported.

This article reports the complete mitochondrial genome of the whitefly *A. shizuokensis*. The whiteflies were collected from *Oxalis corniculata* at Hangzhou, China (30°18′32″N, 120°5′49″E) on 6 July 2020, and deposited at the Institute of Insect Sciences, Zhejiang University, Hangzhou, China. Total genomic DNA was extracted from a female adult using DNeasy Blood & Tissue Kit (Qiagen, Hilden, Germany) and next-generation sequencing was performed at Illumina HiSeq 4000 platform (2 × 150 bp). Raw reads were filtered using Trimmomatic (Bolger et al. 2014) and clean reads were assembled using SPAdes (Bankevich et al. 2012). The assembled mitochondrial genome sequence was annotated with MITOS (Bernt et al. 2013) and tRNAscan-SE (Lowe and Eddy 1997). Some annotations were corrected manually.

The complete mitochondrial genome of *A. shizuokensis* is 16,687 bp in length (GenBank accession number: MT880225), and contains 13 protein-coding genes (PCGs), 22 tRNAs, and two rRNAs. The overall base composition is 33.8% A, 47.0% T, 12.2% G, and 7.0% C, with an A + T bias of 80.8%. All PCGs use ATN as the start codon. *COX1* ends with a single T, and the other 12 PCGs use TAA or TAG as the stop codon. Gene arrangement of the 13 PCGs is identical to that of the giant whitefly *Aleurodicus dugesii* (AY521251) and greenhouse whitefly *Trialeurodes vaporariorum* (AY521265).

The phylogeny of available whitefly species and *A. shizuokensis* was analyzed based on nucleotide sequences of the 13 PCGs. The sequences were aligned using ClustalW in software MEGA (Kumar et al. 2016), followed by eliminating poorly aligned positions and divergent regions using Gblocks (Talavera and Castresana 2007). The phylogenetic relationships were reconstructed using the Bayesian inference and maximum-likelihood methods through MrBayes (Ronquist et al. 2012) and RAxML (Stamatakis 2006). The topology of the phylogenetic trees is consistent with that described in Lei et al. (2019) and *A. shizuokensis* is close related to sugarcane whitefly *Neomaskellia andropogonis* (Figure 1).

**Figure 1.**
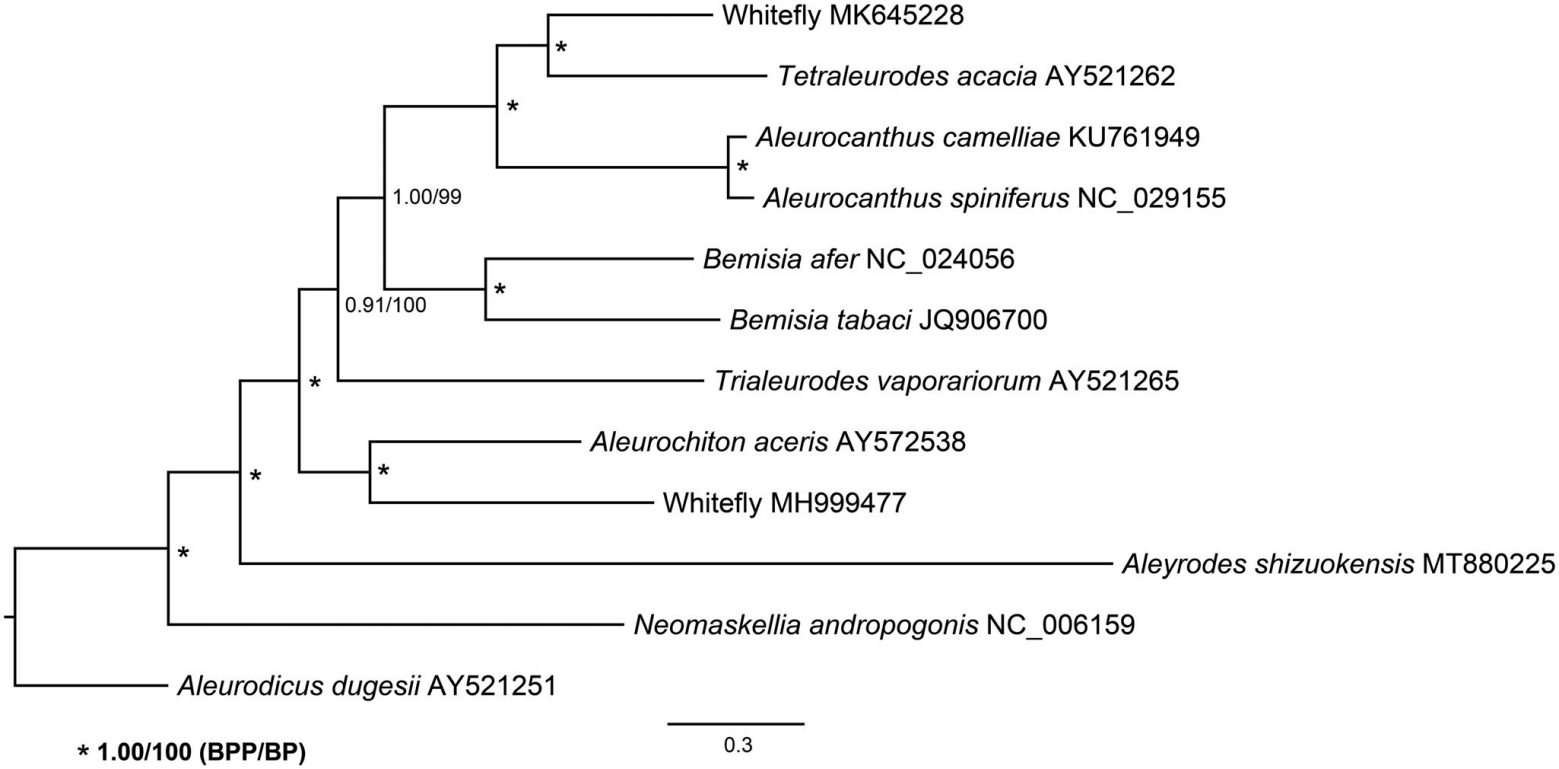
Bayesian inference and maximum-likelihood phylogenetic trees inferred from the nucleotide sequence data of mitogenomic 13 PCGs.

## Data Availability

The data support the findings of this study are openly available in GenBank of NCBI at https://www.ncbi.nlm.nih.gov. The complete mitochondrial genome of *Aleyrodes shizuokensis* for this study has been deposited in GenBank with accession number MT880225. The associated BioProject, BioSample, and SRA numbers are PRJNA681367, SAMN16951332, and SRR13162646.
